# In English Medium Instruction you *can* walk and chew gum

**DOI:** 10.3389/fpsyg.2023.1134982

**Published:** 2023-03-22

**Authors:** ZhaoHong Han

**Affiliations:** Teachers College, Columbia University, New York, NY, United States

**Keywords:** English Medium Instruction, automated processing of natural language, integration of content and language learning, reading and writing, task-based instruction

## Abstract

In English Medium Instruction (EMI), one of the biggest challenges is reportedly the teachers’ own lack of English language proficiency. Helping teachers to improve their proficiency while learning about pedagogy is critical to achieving reasonable success in EMI. This article is contextualized in an English language teacher education program conducted in Tunisia. Specifically, I zoom in on a training task that had trainees reading an academic textbook and posting takeaways on an asynchronous platform over a four-week period. The corpus, comprising 50 journal entries produced by five teacher trainees, was analyzed, first using automated tools for natural language processing and then through human coding, for a combination of quantitative and qualitative perspectives, and with a view to deriving a comprehensive understanding of learning as manifested on multiple levels - psychological, cognitive, and linguistic. Results show impressive learning gains both in content and language. I conclude by discussing the findings and implications for EMI.

## Introduction

In today’s interconnected world, ruled by ever-growing global tendencies toward politics, the economy, climate change, and civil strikes for equity and liberty, English as a *lingua franca* takes on an unprecedented role as a tool of communication. Amid this backdrop, developing countries such as those in North Africa have in recent years started pushing for English as a medium of international communication. Notably, governments have embraced two strategies. One is to provide English as a foreign language (EFL) instruction to young learners. In Tunisia, for example, English is now a mandated part of the national curriculum for primary schools. The other governmental strategy is to adopt English as a medium of instruction in higher education institutions, especially in science, technology, and engineering schools. The push on both of these fronts has meant a greater demand for an instructional workforce that can deliver the expected outcomes. That, however, is not going to be easy. For one, in MENA there has been an ongoing lack of teacher professional development that specifically targets these types of teaching. As a result, teachers who find themselves teaching English either as a subject matter or use English as a medium of instruction for other academic subjects are mostly not ready for the job. For another, there has been a lack of resources available to the teachers, including, but not limited to, a lack of instructional materials, much less an expertise to review and adapt what little is available. But a much bigger challenge is that teachers themselves feel inadequate in their English proficiency. How to address the dual challenge of improving teachers’ English proficiency and at once developing their pedagogical skills is, therefore, a top concern in designing and practicing English Medium Instruction (EMI).

In this article, I draw on a professional development program designed and implemented by Columbia University’s Teachers College in Tunisia. The program was aimed at building capacity for English language education of young learners in Tunisia. Specific to the present purposes, I showcase a training task that has proven effective both to hone teachers’ English language proficiency and to foster their understanding of task-based language teaching. Based on evidence from the Tunisia program (reported in the Study section below) as well as from a previous teacher development program implemented elsewhere (Han, 2018), I argue that it is not only desirable but entirely possible to ‘walk and chew gum at the same time’, meaning to simultaneously promote content and language learning in training English language teachers and by extension, teachers of EMI.

In the sections that follow, I begin by providing some theoretical background for teaching English as a foreign language (EFL), English Medium Instruction (EMI), and task-based instruction (TBI). I then follow up with a brief discussion on the need to reconsider EMI curricular practices. After that, I report on a longitudinal study which tracks five Tunisian trainees’ performance on a reading and writing task. I conclude by discussing the findings and implications for training teachers of EMI.

## English as a foreign language

In academic discussions on teaching and learning an additional language (i.e., additional to the first language), a distinction is frequently made between *second* and *foreign* language teaching and learning, highlighting a difference in the setting where teaching and learning takes place (VanPatten et al., 1987; Gass, 1989). The teaching of English, for one, can be distinguished along that line. If happening in a foreign language environment, the teaching and learning of English is confined to the classroom. But if it happens in a second language environment, both teachers and learners can experience the target language (i.e., the language being learned), in this case, English, both in and out of the classroom. In short, the teaching and learning of English as second language transcends the classroom boundary, whereas as a foreign language, the teaching and learning is bound by it.

This difference is not trivial. The second versus foreign contexts embody different conditions for teaching and learning. Consider the learner’s exposure to the target language (TL) – technically known as “input exposure.” Gass (1989) noted that “[i]t is in the category of input that major differences exist between second and foreign language learning” (p. 42). One can be dubbed an “input-rich” environment, the other “input-poor.” *Second* language teaching and learning comes with greater exposure to input than *foreign* language teaching and learning. This, alone, may have serious consequences for the teaching and learning processes and outcomes (for discussion, see Krashen, 1982; Chaudron, 1988; Ellis, 1988; Gass, 1989; VanPatten, 2004; Loewen, 2014). When there is limited input in the learning environment, learning is made hard by forcing both the teacher and the learner into a precarious and premature reliance on themselves - rather than on the target language - to make sense of how it works. This can be particularly challenging for teachers if they themselves have inadequate proficiency in the target language. Pressed by limited access to sources of target language, these teachers may have no other recourse than resorting to their own, albeit incorrect, intuitions or inventions, such as conjuring up rules for their students to memorize or providing excessive error correction, including treating, inadvertently, non-errors as errors (Stenson, 1975; Kasper, 1982). Guided by such instruction, learners would, at best, wind up with a superficial, pseudo knowledge *about* the TL, not knowledge *of* the TL. The latter, not the former, is what channels fluent, accurate, and appropriate use of the TL (Ellis, 1994; Long, 2015).

Conditions matter. As early as 1967, Corder pinned down the essence of language teaching, stating:

… we cannot really teach language, *we can only create conditions* in which it will develop spontaneously in the mind in its own way. (Corder, 1967, p. 169; emphasis added)

Following this insight, second or foreign language teaching amounts to providing conditions, and while learning develops in its own way beyond the teacher’s control, the conditions may shape the outcomes.

So, what conditions? Five decades of research on second language acquisition (SLA) have boiled them down to four, or INFO as a shorthand (Han, 2007), where “I” stands for input (i.e., exemplars of meaningful use of the target language); “N” for negotiation (i.e., a process of interaction brokering comprehensible input); “F” for feedback (i.e., information on what is not permitted in the TL); and “O” for output (i.e., communicative production of the TL).

The four conditions are essential (Pica, 1987; Long, 1996, 2015; Van den Branden, 1997, 2022; Lee, 2000; Gass, 2013; Ellis and Wulff, 2015; Gass and Mackey, 2015) to language development in any environment, second or foreign. Omission of any of these conditions would jeopardize the quality of learning (see, e.g., Swain and Lapkin, 1995). The obvious truth is that the foreign language classroom typically falls short of these conditions, both in terms of quantity and quality. Thus, the efficacy of foreign language instruction hinges largely on providing and optimizing these conditions.

## English Medium Instruction

English Medium Instruction (EMI) is broadly defined as “the use of the English language to teach academic subjects (other than English itself) in countries or jurisdictions in which the majority of the population’s first language is not English” (Macaro, 2020, p. 534). Simply put, EMI typically happens in an English as a foreign language setting, but the object of instruction is not English *per se* but an academic subject such as Engineering, Biology, Medicine, Physics, and Applied Linguistics.

In an EMI context, the classroom learner, therefore, faces a dual task of learning both the content and the language that encodes the content, and instruction comes down to scaffolding a comprehension of the meaning of the content and, at the same time, cultivating English language proficiency. To wit, both the instruction and the learning revolve around processing and producing meaning (content) and form (language) mappings, importantly, within a disciplinary context. Fundamentally, EMI is about developing an ability in the classroom learner to communicate content in English. And because communication is oral as well as written, EMI is about providing conditions under which the classroom learner can become skilled in both comprehending (through listening and reading) and expressing (through speaking and writing) ideas pertinent to the academic subject, in English.

Models of EMI are many, but four are popular, according to Macaro (2022). The first is called the Preparatory Year Model which has students taking a year or so of intensive English language training before allowing them to join an EMI course or program. This model sees English language proficiency as a prerequisite for EMI. Instantiations of the prerequisite vary between focusing on English for general academic purposes (EAP) and orienting English toward a specific subject matter or discipline. This model potentially requires collaboration between content instructors and English language instructors.

Another model is the Pre-institutional Selection Model whereby students are pre-selected based on their English proficiency for admission to an EMI course or program. The model’s underlying assumption is that students would not need much language help while studying in the EMI program and that teachers would not have to adjust their instruction to different levels of proficiency.

A third model is called the “Institutional Concurrent Support Model.” This model is premised on the assumption that students who have completed secondary schools must have developed a certain level of proficiency in English and therefore are ready for EMI. Thus, anyone who has completed their secondary education qualifies for EMI. Different from the Pre-institutional Selection Model, however, here students may receive remedial EAP instruction, which requires an understanding on the content teacher’s part of the students’ linguistic needs and, likewise, an understanding on the English language teacher’s part of how to meet the needs such as the need to learn the specific academic genre and the corresponding language.

The fourth model to mention here is the “Multilingual Model” which essentially offers flexibility in the use of English in teaching the content of a subject matter. Variations in the medium of instruction – English or any other language - are allowed not only in a given session but also across sessions. For example, some sessions can be taught in the L1 (e.g., Arabic) and some in English.

In addition to these curricular options, EMI is non-monolithic in another way. Macaro (2020, 2022) made a useful distinction between “hard-core EMI” and “soft EMI.” The so-called “hard-core EMI” concerns “policy-led” decisions on teaching the subjects which can otherwise be taught in the L1 - Physics, Mathematics, Engineering, Geology, to name but a few. Soft-EMI, on the other hand, is “language-led.” Examples are Applied Linguistics, TESOL, International Business, and Translation Studies, academic subjects naturally involving the use of the English language. Logically, soft-EMI should be easier to conduct than hard-core EMI, which, typically mandated by an educational policy at a national or institutional level, would require a greater synergy between content instruction and foreign language pedagogy or a greater collaboration between content and language instructors.

In his overview of EMI research, Macaro (2022) spotlighted a number of ongoing questions, one of which is: *What kind of professional development do EMI teachers need, and what barriers are imposed?* Our discussion so far has made clear that in a foreign language setting, the teacher’s English proficiency is pivotal to students’ learning. EMI in a foreign language setting doubles the challenge if neither the language instructor nor the content instructor has adequate proficiency to teach the content through English (Costa and Coleman, 2013; McKinley et al., 2022). Not much learning is likely to result. How to improve the teacher’s own English proficiency should, therefore, be central to EMI professional development (Borg, 2018; see, however, Bradford, 2019), on par with the importance of educating teachers on pedagogy.

The Macaro (2022) synthesis illuminates a cause for concern. Much of the research (and for that matter, discussions) on EMI has taken place in a silo, so to speak; researchers seemed unaware of the more general field of SLA research, though it is ostensibly changing. For example, one of the questions Macaro (2022) raised about current EMI was: *how should pedagogy change in an EMI setting with a particular focus on interaction?* This calling for attention to interaction in the classroom was a response to the reality that most EMI instruction has largely been delivered through lectures (see, e.g., Hellekjaer, 2010; Costa and Coleman, 2013). The emphasis on interaction was likely inspired by the Interaction Hypothesis (Long, 1996) or informed by SLA empirical reports on the acquisitional benefits of interaction. Overall, however, few such insights have been incorporated in EMI and research.

Still, extant EMI studies, mostly surveys to ascertain either teachers’ and students’ perceptions of EMI (see, e.g., Hamid et al., 2013; Macaro and Akincioglu, 2017; Macaro et al., 2020) or the scope and distribution of EMI programs in a given region (see, e.g., Costa and Coleman, 2013), have collectively put a spotlight on teachers’ lack of proficiency and lack of pedagogical training – both part and parcel of teacher competence – as primary barriers in EMI.

Breaking down the barriers, in my view, would require greater interface with SLA. The SLA literature offers a wealth of insights which EMI can and should draw on to inform its curricular designs and teacher professional development. Investigating these insights in the context of EMI may lead to breakthroughs in conceptualizing and implementing EMI, ultimately enabling strides toward greater efficacy.

## Reconcpetualizing EMI curriculum and teacher development

For EMI curriculum development, useful insights can be gained, for example, from Hulstijn’s (2015) model of language proficiency, a model originally developed for charactering and understanding native speaker proficiency. The model differentiates between two types of proficiency, Basic Language Cognition (BLC) and High Language Cognition (HLC). BLC covers the largely implicit, unconscious knowledge in the domains of phonetics, prosody, morphology and syntax. HLC, an extension of BLC, concerns sophisticated language use of spoken and written language, for instance, the use of low-frequency lexical items and uncommon morpho-syntactic structures. On a continuum, both BLC and HLC have a core and peripheral dimension: The core from BLC to HLC is, respectively, linguistic knowledge and knowledge of communicative use of language. The peripherals run the gamut of interactional ability, strategic competence, metalinguistic knowledge, and knowledge of discourse types. Linking this model of proficiency to EMI, the implication is that fundamentally EMI is about cultivating proficiency in HLC, the ability to use the language communicatively. However, because HLC subsumes BLC, it would be impossible to cultivate HLC without also developing learners’ BLC. Predicating on this reasoning, Han (2016) advocated that English for Specific Purposes (ESP) should adopt a more integrated curriculum with HLC as a point of departure, rather than BLC as a necessary first step (as implicated in the EMI models discussed above).

For EMI professional development, especially for improving EMI instructors’ own English proficiency as well as their students’, the SLA usage-based learning paradigm is uniquely relevant that places a dual premium on the quantity and quality of the linguistic environment and the learner’s experience with the target language input (see, e.g., Bybee, 2008; Ellis and Wulff, 2015; Luk and Rothman, 2022). Given its overarching goal of developing learners’ functional competence in English vis-à-vis an academic subject, it only stands to reason that EMI should provide usage-based instruction (Verspoor, 2017; Han, 2020; Lowie et al., 2020), as should the training of EMI teachers if improving teachers’ English proficiency is part of the goal.

In line with the usage-based framework, SLA research over the past three decades has actively explored a pedagogical framework called task-based instruction (TBI). TBI champions the use of communicative tasks as a vehicle of development of communication skills – listening, speaking, reading, and writing (see, e.g., Ellis, 2003; Willis and Willis, 2007; Long, 2015; Van den Branden, 2022). Research has, *inter alia*, found that TBI is well suited for developing learners’ communicative proficiency on both cognitive and affective accounts. On the cognitive side, task-mediated instruction creates affordances (a) for active and iterative engagement with rich, authentic target language input, (b) for negotiation of meaning and form, (c) for contextualized feedback, and (d) for meaningful production of output – the essential INFO conditions as referenced above. On the affective side, task-based instruction is motivating in that it is ecologically valid with strong connections to real-world use of language while being learner-centered, and it encourages and fosters learner agency and autonomy. Accordingly, it does not come as a surprise that TBI has been widely embraced in second and foreign language instruction across the globe, especially in ESL and EFL teaching.

Despite that TBI has been extensively researched, even earning the reputation of “a researched pedagogy” (Van den Branden et al., 2018), an as yet underexplored theme of TBI is whether or not it is applicable to EMI, let alone EMI teacher professional development. Theoretically, TBI - by virtue of its meaning primacy and its contextualized attention to language – should be appropriate, if not ideal, for ESP, EAP, and EMI (see also Van den Branden, 2022). Empirically, research on task-based EMI (TEMI) is on the horizon, with emerging evidence that it is possible to walk and chew gum at the same time – simultaneously facilitating content and language learning.

## Walking and chewing gum at the same time

In a study reported in *Annual Review of Applied Linguistics*, Han (2018) implemented a closed-loop strategy (Adams, 1971) - whereby the content and procedure of training are integrated in the classroom - in a foreign language teacher education program conducted in the U.S. that trained teachers of Chinese. The content in the said case was task-based language instruction, delivered through English, hence EMI, but the training procedure was also task-based. Simply put, the closed-loop strategy was about using task-based pedagogy to facilitate the learning of TBI, with a two-pronged goal of helping trainees develop a conceptual understanding of TBI and at the same time creating affordances for trainees to improve their own English language proficiency. One of the training tasks employed was writing a reading journal over the course of 10 weeks. Trainees, who were native speakers of Chinese, read Willis and Willis (2007), an introduction to TBI. The book is comprised of 10 chapters (see Table 1). Upon reading each chapter, trainees wrote about their takeaways in English.

**Table 1:** Chapter titles in Willis and Willis (2007).

Chapters	Titles
Chapter 1	The basis of task-based approach
Chapter 2	Task-based sequences in the classroom
Chapter 3	Tasks based on written and spoken tasks
Chapter 4	From topic to tasks: listing, sorting, and classifying
Chapter 5	From topic to tasks: matching, comparing, problem-solving, projects, and storytelling
Chapter 6	Language focus and form focus
Chapter 7	The task-based classroom and the real world
Chapter 8	Adapting and refining tasks: seven parameters
Chapter 9	Designing a task-based syllabus
Chapter 10	How to integrate TBT into coursebooks and other frequently asked questions

The Han (2018) study was guided by this question: To what extent was task-based learning beneficial to improving trainees’ understanding of TBI as well as their linguistic ability to express their thoughts (i.e., language learning)? To that end, the journal entries produced by three participants – representing a spectrum of teaching experience and prior training in foreign language pedagogy - were subject to a multi-dimensional, mixed-methods analysis of (a) the cognitive and emotional processes, (b) the conceptual learning of TBI, and (c) the linguistic gains. Results showed:

tangible evidence in support of a fundamental tenet of TBLT [task-based language teaching]—though still understated in the current literature—*that it can result in both content and language learning*, and [the study] demonstrated it for the first time in a non-language-learning arena.

Han went on to say:

This [finding] opens up not just one additional avenue, that of foreign language teacher training, for investigating the potential of TBLT but also, conceivably, multiple avenues, so long as the contexts are content-based and involve L2 learners or users—content-based instruction in K–12 schools, vocational training for immigrants, and the like. (p. 183)

The study reported below largely replicated this finding.

## The study

The present study was contextualized in a foreign language education program designed for teachers of young learners of EFL (TEYL) in Tunisia. In one of the program modules taught by the author, participants received training in TBI, and performed, *inter alia,* a longitudinal reading and writing task, which was identical to the reading journal task described in Han (2018). The research question guiding the study was also the same, aiming at capturing content and language learning.

### Participants and data

Participants were five teachers,^1^ pseudo-named K, N, J, Z, and S, who, at the time of attending the TEYL training program, were teaching English at various higher education institutes in Tunisia. The training program comprising four modules was conducted remotely, spanning one calendar year. While attending Module 4, the participants were introduced to TBI, and concurrently, as an assignment, they in 4 weeks read Willis and Willis (2007) and wrote about their takeaways following the reading of each chapter. The five sets of 10 journal entries, therefore, comprised the corpus for the present study.

### Data analysis

As in Han (2018), the corpus of journal entries was subjected to analysis using a machine-human hybrid approach. Specifically, the corpus underwent two rounds of analysis, first automated and then manual. The goal was to achieve both a quantitative and a qualitative understanding of the data. The automated analysis, yielding quantitative results, was done at two levels: content and language. Two robust text-based computational programs for natural language processing were employed as analytic tools: Linguistic Inquiry and Word Count (LIWC) for content analysis and the Lexile® framework for language analysis. LIWC measures, and produces indexes for, the social, cognitive, and psychological dimensions of writing (Pennebaker et al., 2015). The Lexile® framework (MetaMetrics, 2015), on the other hand, assesses semantic and syntactic complexity, with higher scores denoting higher syntactic and semantic complexity or greater sophistication of language use. Lexile scores can range from below zero to above 2000 L.

Specific for the present study, the corpus was analyzed using LIWC for emotional tone, analytic thinking, and authenticity, the resulting scores indexing the psychological and cognitive dimensions of content learning. The same corpus was then analyzed using the Lexile® framework for language learning. To illustrate, consider two paragraphs in Sample 1.

#### Sample 1

- In the approach, the grammar is viewed as a vital point if we want to make what you want to say easily understand by our listeners. it is possible that we can understand most of the broken sentences that spoken by Chinese language learners, but later, when the learners want to express more complex meanings, they will feel helpless and confused if they do not have a good command of grammar! So, in the book, they give out two possible starting points for teaching language, i incline toward the first one “to see meaning as a starting point for language development, and to see form as developing from meaning.” after all, the vocabulary is the key to the meaning, the meaning is the key to the communication.
- as my English learning experience, grammar is always the core part of the curriculum. Yes, grammar and form are important in language learning, yet in the chapter, they illustrate “why not start with grammar” and give out an example “Yes/No challenge.” the game looks easy but it is “extremely difficult” to focus on the accuracy of what you are going to say. Be- sides, once learners focus more on the form, they probably lose the fluency and cannot convey their meaning confidently because forms restrict their thoughts.

Table 2 summarizes the results from the automated analyses.According to the LIWC analyses, the two paragraphs achieve very different scores on analytical thinking (95.9 vs. 83.4), with (a) exhibiting greater formal, logical thinking than (b), as evident in (a) deploying expressions like “if,” “it is possible,” “but,” “so,” “after all,” and (b) fewer such expressions. However, in terms of authenticity, (a) shows less originality than (b) (31.5 vs. 47.9). This is seen in (b) being about one’s own thoughts rather than about the pedagogical proposal discussed in the book chapter, as in (a). Similarly, (a) carries a lower emotional tone than (b) (39.9 vs. 69.1)—where emotions are encoded by words such as “easy,” “extremely difficult,” “lose,” and “cannot.”

**Table 2:** Results of automated analyses of sample 1.

Domains	Tools	Measures	Paragraph (a)	Paragraph (b)
Content	LIWC	Analytical thinking	95.9	83.4
		Authenticity Emotional	31.5	47.9
		Tone	39.9	69.1
Language	Lexile	Lexile score	1390L	1220L

The Lexile measure of language use shows that (a) is more sophisticated than (b) (1390L vs. 1220L), meaning that (a) features greater syntactic and lexical complexity than does (b).

Thus, the automated analyses presented a nuanced picture of content learning and language use.

In an effort to adequately trace participants’ learning of content and language, in the present study the automated analyses were then augmented by qualitative analysis of select writing samples, carefully inspected for discourse evidence to substantiate the automated results.

## Results

Table 3 displays the average scores results from LIWC and Lexile analyses for the five participants.

**Table 3:** LIWC and Lexile mean scores for all five participants.

Participants	LIWC				The Lexile® framework			
	Positive tone	Negative tone	Analytic thinking	Authenticity	Lexile measure	Mean sentence length	Mean log word frequency^*^	Word count
K	1.133	0.238	0.8	0.68	1325L	22.96	3.36	262
N	2.199	0.514	0.8	0.66	1418L	28.39	3.42	424
J	1.915	0.464	0.92	0.4	1505L	30.21	3.25	353
Z	2.267	0.29	0.84	0.53	1458L	27.24	3.22	410
S	1.614	0.355	0.91	0.35	1479L	30.58	3.21	179

^*^A measurement of semantic difficulty, the higher the frequency the lesser the semantic difficulty.

**Figure 1:**
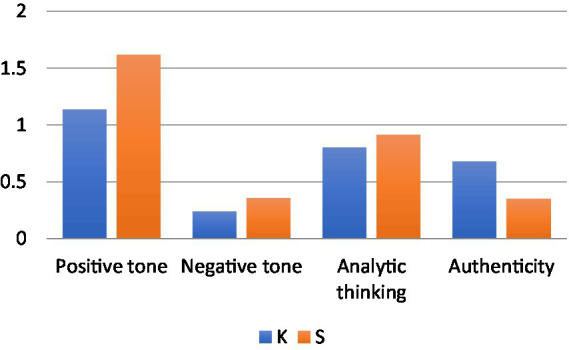
LIWC analyses of content: K vs. S.

A quick inspection of the scores displayed in Table 3 reveals that all five participants performed differently on the reading and writing task, with distinct LIWC and Lexile scores. A close look at the mean scores shows that the individuals’ journal entries displayed different levels of positive and negative tone, analytic thinking (i.e., processing the information content of the chapter), and authenticity (i.e., pursuing own thinking). For instance, participant K, compared to participant S, had on average a much lower positive tone (1.133 vs. 1.614), lower negative tone (0.238 vs. 0.355), lower analytic thinking (0.8 vs. 0.91), but much higher authenticity (0.68 vs. 0.35). K’s and S’s Lexile scores were different as well. K’s total Lexile score was lower than S’s (1325L vs. 1479L), but his journal entries had a higher average word count than S’s (262 vs.179). In terms of syntactic complexity, however, K’s average sentence length was lower than S’s (22.96 vs. 30.58 words). Yet in terms of semantic complexity, K’s was slightly higher than S’s (3.36 vs. 3.21). Figure 1 gives a visual comparison of K’s and S’s average performance on the content indexes as measured by LIWC.

Figure 1 shows that relative to S, K was on the whole less emotional about the content of the chapters, performed less reasoning when reading them, but had more original thinking about the chapters.

**Figure 2:**
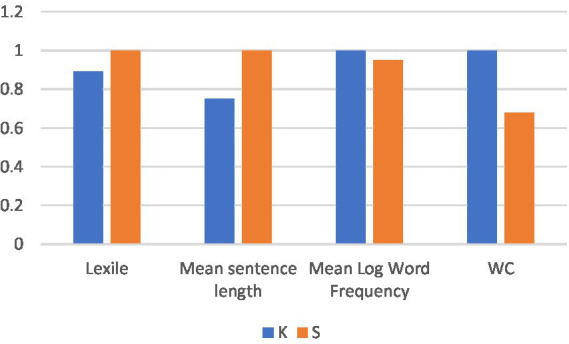
Lexile analyses of language: K vs. S.

Figure 2 gives a visual display of K’s and S’s average performance as measured by Lexile. In spite of the fact that K on average wrote longer journal entries than S (see Table 1), his overall syntactic and semantic complexity were lower than S’s.

It is clear from Table 3 that there were no two participants completely alike, and that was not surprising, given that the participants had each brought different resources – cognitive, affective, linguistic, and experiential - to their interaction with the text. To ascertain whether content and language learning have taken place in the participants, it is crucial to find out how they individually responded to each chapter and how they individually fared over the course of reading and writing about the 10 book chapters.

In the interest of space, here below I provide an in-depth analysis of K’s 10 journal entries. The choice of K is deliberate because of his comparatively lower average score on the Lexile measure (see Table 2). For the sake of argument, I will proceed from the automated results on K’s content and language to a qualitative scrutiny of a selection of his writing samples.

**Figure 3:**
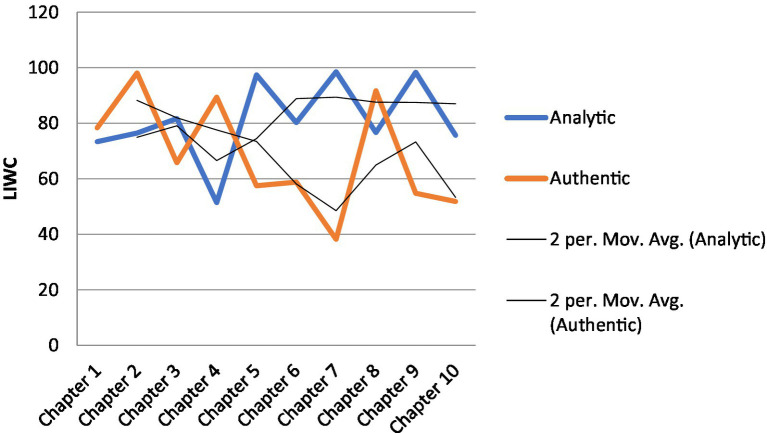
K’s analytic thinking and authenticity across 10 chapters.

**Figure 4:**
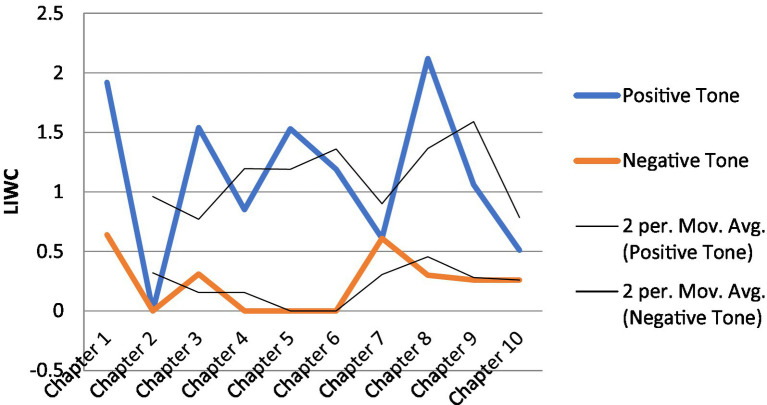
K’s emotional tone across 10 chapters.

Table 4 gives an overview of K’s LIWC scores across the 10 chapters he read and wrote about. Figures 3, 4 provide a visual display of K’s trajectories as measured by LIWC.

**Table 4:** K’s LIWC scores across 10 chapters.

	LIWC	C1	C2	C3	C4	C5	C6	C7	C8	C9	C10
K	Analytic thinking	73.36	76.47	81.71	51.39	97.46	80.22	98.54	76.64	98.37	75.64
	Authenticity	78.33	98.12	65.75	89.41	57.46	58.8	38.18	91.71	54.79	51.79
	Positive tone	1.92	0	1.54	0.85	1.53	1.19	0.61	2.12	1.06	0.51
	Negative tone	0.64	0	0.31	0	0	0	0.61	0.3	0.26	0.26

Figure 3 shows K’s scores of analytic thinking and authenticity for his writings on the 10 chapters of Willis and Willis (2007). The scores fluctuated throughout. The moving average trend lines indicate that K started out with higher authenticity than analytic thinking (see Chapter 1), but mid way (see Chapter 4), the trend began to flip such that his analytic thinking became higher than his authenticity, suggesting that across time, he became more drawn to the content of the chapters and was better able to relate to the authors’ view points.

Likewise, K’s emotional tone fluctuated as his reading and writing proceeded. Figure 4 shows changes in K’s emotional tone with moving average trend lines. One noticeable thing about the trajectory was that while K’s positive tone was always higher than his negative tone, the discrepancy between the positive and the negative became wider around Chapter 4, with the positive much larger than the negative. This corroborates the “flip” noted above between K’s analytic thinking and authenticity, suggesting that K had a transformation in his understanding of, and disposition toward, TBI. Of note also is that the gap between the positive and the negative started to narrow from Chapter 7 onward until the two came very close (see Chapter 10), bookending the journey of reading and writing with a balanced understanding of TBI.

In order to substantiate the automated results, let us look at three samples (samples 2–4) of K’s journal entries, respectively, on chapters 4, 7, and 10.

### Sample 2: K’s journal entry on Chapter 4

Tue Sep 14, 2021 at 11:07 am

While reading through the pages of this chapter I was amazed by the number of clear examples and illustrations accompanying the author's account of the approach. I have been reading quickly because that account seemed to me quite familiar since much of what has been detailed is not new for me, some of it, I read about in past occasions and some other heard from many ELT inspectors. This doesn't mean that I learned nothing from this chapter, quite the opposite, I learned a lot by what it has confirmed in me. I was reading and jumped o some parts, I did that because I was reading for specific information to a question hanging in my head: where is reading and writing to all this? All that s been said in the chapter, as you might have noticed, was about task sequences serving as input for oral and aural language production. Only later in the last pages precisely where I found some release and got my answer. What is it? Yes the listing, classifying and ordering task types that formed the bulk of the lessons can be followed up a reading text the topic of which was previously introduced. Writing too has its place, since 'it s possible to ask them [learners] to brainstorm in writing, either individually or in pairs, and then to present their ideas in writing' (chapter 4).

### Sample 3: K’s journal entry on Chapter 7

Sun Sep 26, 2021 at 6:09 am

Willis and Willis in this chapter (7) get to the bottom in their TBLT account. The depth lies in highlighting what makes an artificial task different from a real world one. Furthermore, they pointed to the distinctions between a spontaneous spoken discourse and an electronic one. An artificial task or a ‘fake task’ as Dr Han (previous session) has labeled it, deprives the learner from what the real world tasks can offer. They lack the situational authenticity which is a feature inherent in real world tasks; language is formal and contrived going counter to the characteristics of a genuine task. By the same token, in particular, at the spoken discourse level, L2 teachers are expected, in Willis and Willis views, to pay attention to some discourse features, such as false starts, vague language, the use of fillers, the use of ‘tails’… and bring their learners’ attention to them, and more importantly to raise their awareness to the social/cultural dimension encoded in the language.

### Sample 4: K’s journal entry on Chapter 10

Tue Oct 5, 2021 at 4:31 pm

I want to begin this reflection by the fact that Willis and Willis in this chapter (10.2.1) confirmed a conviction I was holding taht some activities are tasks, i.e, the difference is in the name. In our Tunisian context, and especially in some textbooks many activities are tasks in fact, in the sense that they satisfy Willis and Willis conditions laid out in this book, but simply they bear the name of “ACTIVITY”.

Though Willis and Willis probably have not surveyed Tunisian teachers on their experiences with TBLT as they did with other teachers from different countries, the list of problems (figure 10.1) they present in this book echoes much of what many of my English language colleagues in Tunisia believe, as regard TBLT. Personally I find the following points as real problems:

- exams are not task based
- learner use minimal language and leave much of the class work to the teacher
- some of the old textbooks do not have tasks in.

Furthermore, as I proceeded in the chapter, I found the following observation from the authors surprising; **‘enacting a conversation’ is not a task**. Before I read this observation I thought the opposite is true. In Willis and Willis words ‘they may have meaning potential, but they are not primarily concerned with meaning’ (10.2.1), simply put, they are form-focused activity.

I also found, in this chapter (10.7), that the tolerance for some use of the L1 quite natural, as TBLT proponents allow for it. But what sounds extreme, is the deliberate use of the L1 in a whole activity. This is what Heidi Vande Voort in Korea and Annamaria Pinter in Hungary (2006) did with their advanced level students, of course they may have their reasons. I think this would be quite unwelcome from ELT inspectors in the Tunisian classrooms.

Last, although in the previous sessions with Dr. Han we didn’t discuss how assessement is conceived in TBLT, I found an interesting hint to that in (10.13). The **Exams** or **tests** in a TBLT approach should reflect **communicative assessment criteria** (appropriacy, fluency …). What is meant perhaps by appropriacy and fluency is that language input (texts written or in audio formats) should reflect those criteria, but **the question that remains unanswered is how can 'grammar points' be assessed in the light of these criteria?**

The three samples offer a window on the dynamic process of K’s interaction with Willis and Willis (2007), and importantly, they provide a qualitative perspective on the LIWC results. K’s writing on Chapter 4 (see Sample 2 above) indicates that he was positive about his learning from the chapter, appreciating the “examples and illustrations” given therein. But his reading was largely guided by his *own* questions. For instance, he looked specifically for information on reading and writing but did not find it until about the end of the chapter. He then reasoned for himself why the information was given late in the chapter. All this explains why K scored lower – in fact the lowest of his 10 journal entries - on analytic thinking but much higher on authenticity (51.39 vs. 89.41).

Compared to his writing on Chapter 4, K’s writing on Chapter 7 (see Sample 3) was far more positive. He was impressed with the depth of the chapter, citing an insightful distinction made by Willis and Willis between an artificial task and a real-world task. By this time, K showed an ability to integrate an ongoing class discussion with what he was reading in the chapter, invoking concepts such as “situational authenticity” to enrich his own understanding of the distinction made by Willis and Willis. He was indeed very analytic, much more so than being himself or being authentic (98.54 vs. 38.18).

K’s writing about Chapter 10 (see Sample 4) demonstrates his content learning reaching a whole other level. This intensely engaging piece features K actively interacting with the information presented in the chapter, confirming or disconfirming his own prior understanding of concepts of TBI, making connections with the ongoing class discussion as well as with his own teaching context in Tunisia, critiquing Willis and Willis’s recommendation on deliberate use of the L1 in TBI, all while continuing to search for answers to his own questions. K ended his writing not with a sweeping statement, as many would do, but with a question that was still lingering in his mind, signaling that his learning about TBI did not stop with this chapter or with the entire Willis and Willis book, but was to continue. The LIWC scores for this entry indicate a balance between his analytic thinking and his authenticity (75.64 vs. 51.79).^2^

More profoundly, the three samples of K’s journal entries illustrate a developmental trajectory of content learning, marked by a shift from an initially modest enthusiasm for the Willis and Willis treatise of TBI to a growing interest and then to an extraordinarily high-level engagement with it. K’s last journal entry reflects a top-notch understanding of TBI that is both abstract and tangible, both macro and micro, and both global and local.

**Figure 5:**
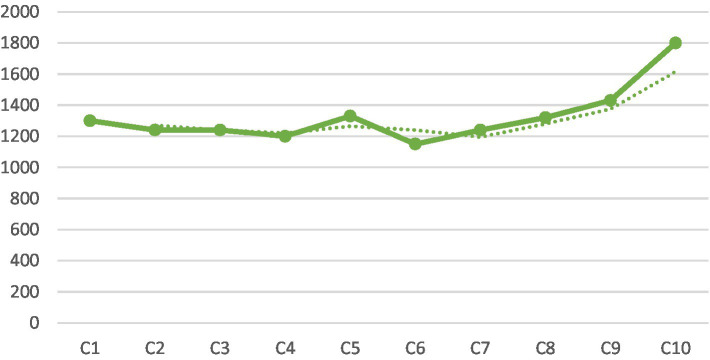
K’s Lexile scores across 10 chapters.

Given the amount of content learning occurring in K, it would only be natural if there was language learning happening alongside (Robinson, 2001). Table 5 gives K’s Lexile scores for the 10 chapters he read and wrote about, and Figure 5 provides a visual display of K’s scores on the Lexile measure for his writings on all 10 chapters.

**Table 5:** K’s scores across 10 chapters from the Lexile analyses.

	Lexile measure	Mean sentence length	Mean log word frequency	Word count
C1	1300L	21.71	3.19	152
C2	1240L	23	3.49	69
C3	1240L	22.86	3.56	320
C4	1200L	23.2	3.62	233
C5	1330L	21.75	3.13	261
C6	1150L	20.75	3.54	332
C7	1240L	20.38	3.26	163
C8	1320L	23.43	3.3	328
C9	1430L	26.71	3.22	374
C10	1800L	25.8	3.32	387

Figure 5 shows a clear upward trend starting from K’s journal entry on Chapter 7. This is coterminous with a qualitative change in K’s content learning as noted above. Notice that K’s last journal entry (on Chapter 10) achieved a high score of 1800 L, indicating that his language use was highly sophisticated. Put differently, there is consistency between K’s content learning and language use. This confirms a prediction made by Robinson’s (2001) Cognition Hypothesis, that when the thoughts are complex the language is sophisticated (see also discussion in Han and Kang, 2018).

## Discussion and conclusion

The present study replicated a major finding from Han (2018), namely that it is possible to simultaneously stimulate content and language learning in an EMI environment. The current study, zooming in on five participants in a foreign language teacher education program, shows that the reading journal task provided ample affordances for learning about TBI (i.e., content) and for honing language use. An in-depth analysis of one of the participants, K, offered critical insights on the process of learning, especially how a focus on meaning comprehension (i.e., reading) led to incidental learning of the English language. As a result of his increasing conceptual engagement with the content of the Willis and Willis (2007) book, K’s language use (i.e., writing) grew markedly more sophisticated.

The dual success of the reading and writing task did not, however, come from participants performing the task once; rather, it came from *their iterative engagement with the task over an extended period of time*. It was the repeated experience with the task that facilitated content learning and language learning: what was initially incomprehensible could become comprehensible, and what was initially a non-salient linguistic expression could become perceptually salient.

Repeated reading as a reading method has been amply validated in language development research and has been shown to be effective at improving reading comprehension and fluency (see, e.g., Moyer, 1982). A study by Han and Chen (2010) demonstrated the efficacy of repeated reading in improving reading comprehension and boosting vocabulary acquisition. When repeated reading and repeated writing are in tandem, as is true of the task at hand, the affordances for content and language learning would only multiply. This is because a unique set of conditions has been created - by virtue of coupling reading and writing – for “language mining” (see discussion in Han, 2020). The learner can readily and directly appropriate in their writing linguistic expressions they have encountered through their reading. More profoundly, from a psycholinguistic perspective, a virtuous processing cycle is created such that input begets more output and output, in turn, begets a great desire for more input.

And there is one other catch. The repetitiveness of the reading and writing task, which in other circumstances might have induced boredom and fatigue, was in the present study enhanced by the type of the reading material. The participants read a monograph with 10 chapters on a variety of sub-topics related to TBI. The authors’ consistent writing style throughout could be helpful to the reader, as well as the fact that all chapters revolve around the same theme, stacked in a logical sequence, with one chapter paving the way for the next. All of this combines to have kept participants engaged with the reading. In a way, the reading and writing task enacted a variation of “narrow reading,” which Krashen (1981, 2004), among others, has promulgated. Narrow reading, like repeated reading, comes with rich affordances for language learning, such as lexical frequency and morphosyntactic similarity, and has been found to be effective at fostering language acquisition (see, e.g., Schmitt and Carter, 2000).

In essence, both repeated reading and writing and narrow reading and writing are instantiations of usage-based learning, a learning theory discussed earlier in this article. The theory underlines the importance of letting the language learner experience the target language through usage.

The positive findings reported in this article as well as elsewhere (Han, 2018) bode well for EMI, including EMI teacher professional development. The findings demonstrated unequivocally that walking and chewing gum at the same time – promoting content and language learning in the same breath – is not only desirable but also feasible.

The present study was conducted in what Macaro (2022) has referred to as a soft-EMI environment – a teacher education program on TEYL in Tunisia – where the use of the target language English is not only natural but necessary. Nonetheless, the pedagogical conditions created in this environment are generalizable to hard-core EMI. Fundamentally, as discussed earlier in this article, EMI is about creating conditions conducive to content and language learning, about mitigating the problem of lack of input generally plaguing EFL teaching, and about boosting teachers’ English language proficiency. The trick, of course, lies in doing all of the above simultaneously, rather than sequentially as seen in current EMI models.

EMI has a silver lining if it is to break its status quo: The field of SLA has already produced extensive and robust insights on which EMI can and should draw.

## Data availability statement

The raw data supporting the conclusions of this article will be made available by the authors, without undue reservation.

## Ethics statement

Ethical review and approval was not required for the study on human participants in accordance with the local legislation and institutional requirements. The patients/participants provided their written informed consent to participate in this study.

## Author contributions

The author confirms being the sole contributor of this work and has approved it for publication.

## Conflict of interest

The author declares that the research was conducted in the absence of any commercial or financial relationships that could be construed as a potential conflict of interest.

## Publisher’s note

All claims expressed in this article are solely those of the authors and do not necessarily represent those of their affiliated organizations, or those of the publisher, the editors and the reviewers. Any product that may be evaluated in this article, or claim that may be made by its manufacturer, is not guaranteed or endorsed by the publisher.

## Footnotes

^1^The five teachers all completed the reading and writing task, while their peers in the training cohort fell short, due to various reasons, including COVID.

^2^The gap between the score for analytic thinking and the score for authenticity offers a useful window on the extent to which the writer hews to the information presented in a given chapter versus to themselves – their own thoughts or experience. In the case of the three sample journal entries, the gaps are as follows: −38.02 (authenticity>analytic thinking) for chapter 4; 60.36 (analytic thinking>authenticity) for chapter 7; and 23.85 (analytic thinking>authenticity) for chapter 10.
